# Chloe for COVID-19: Evolution of an Intelligent Conversational Agent to Address Infodemic Management Needs During the COVID-19 Pandemic

**DOI:** 10.2196/27283

**Published:** 2021-09-21

**Authors:** Sophia Siedlikowski, Louis-Philippe Noël, Stephanie Anne Moynihan, Marc Robin

**Affiliations:** 1 Dialogue Health Technologies Inc Montreal, QC Canada

**Keywords:** chatbot, COVID-19, conversational agents, public health, artificial intelligence, infodemic, infodemiology, misinformation, digital health, virtual care

## Abstract

There is an unprecedented demand for infodemic management due to rapidly evolving information about the novel COVID-19 pandemic. This viewpoint paper details the evolution of a Canadian digital information tool, *Chloe* for COVID-19, based on incremental leveraging of artificial intelligence techniques. By providing an accessible summary of *Chloe’s* development, we show how proactive cooperation between health, technology, and corporate sectors can lead to a rapidly scalable, safe, and secure virtual chatbot to assist public health efforts in keeping Canadians informed. We then highlight *Chloe’s* strengths, the challenges we faced during the development process, and future directions for the role of chatbots in infodemic management. The information presented here may guide future collaborative efforts in health technology in order to enhance access to accurate and timely health information to the public.

## Introduction

In the early stages of a global health crisis caused by a novel disease such as COVID-19, misinformation tends to propagate rapidly due to the absence of easily accessible and reliable information from authoritative sources [1]. According to the World Health Organization, controlling the circulation of information about a novel disease requires infodemic management, defined as the application of “evidence-based interventions that bring understandable, localized, evidence-based information to citizens and drive positive health-seeking behaviour” [2]. At the onset of the COVID-19 pandemic in Canada, however, public health hotlines were overwhelmed with calls, impeding the infodemic management process. For example, on March 11, 2020, Quebec’s provincial health information 811 phone line received 16,840 calls compared to an average of 6000 calls managed on a typical day [3].

In recent years, chatbots have emerged as a promising means to support public health efforts by easily increasing access to accurate, tailored, and free information to millions of people at once [4]. Leveraged with artificial intelligence (AI) tools, chatbots are intelligent conversational agents that result from the application of natural language processing techniques to analyze speech combined with an engine that is trained to provide human-like responses [5].

The recent surge in chatbot use across industries and business functions such as customer service, sales, marketing, and automation of internal processes stems from an evolution in the way humans communicate [6]. The current digital age, in which most individuals have access to a mobile phone and are accustomed to holding multiple conversations at once through short online interactions, represents an ideal environment for chatbot development [6]. Since the onset of the COVID-19 pandemic, chatbots are increasingly being developed to efficiently deliver evidence-based information about COVID-19 to the public [4]. Despite these advances, the possibility of chatbots causing harm by communicating inaccurate information to users warrants ongoing monitoring of chatbot quality and safety [7].

The development of Dialogue Health Technologies Inc’s chatbot *Chloe* represents a successful Canadian cooperation among health, technology, and corporate sectors, in order to assist the public during the pandemic by addressing their questions and concerns about COVID-19 [8]. Founded in 2016, Dialogue is a leading provider of virtual health care in Canada, offering its services exclusively to Canadian organizations through an online care platform using technologies developed in-house. This care platform consists of an interface in both desktop and mobile application formats that connects members to a care team consisting of health providers and coordinators. Our experience with chatbots in health care includes the design of various AI-enhanced bots used to collect and intuitively interpret patient information in order to determine urgency, appropriateness for virtual care, and the type of provider required. These chatbots were built by combining medical knowledge with state-of the art algorithms and are continuously monitored, updated, and trained to enhance their effectiveness and efficiency, including bot-to-human handoff when required [9].

*Chloe* was initially conceived to integrate within the Dialogue care platform as a way to help manage questions about COVID-19 from our clients but was quickly converted into a stand-alone digital application in order to be deployed as a free tool for the general public. After Health Canada confirmed the first Canadian case of COVID-19 on January 27, 2020, our medical operations and customer success teams noted an increased flow of questions from members and organizations across the country about this novel disease, looking to us as a health care company for guidance. The urgent need to slow the spread of the virus and provide large-scale access to free information about COVID-19 from authoritative sources, as well as the desire to assist flooded provincial health information phone lines in answering common questions from Canadians, prompted us to pursue an AI approach.

We launched the chatbot *Chloe*, named after Dialogue’s first nurse, on March 9, 2020. *Chloe* was designed to provide free, up-to-date information to all Canadians on COVID-19 from trusted Canadian authorities, following federal, provincial, and territorial regulations based on each user’s location. To ensure transparency of our technological approach, we rendered the online service and content management system open-source. *Chloe* was designed following best dialogue management principles [10] and built with Rasa, a state-of-the-art open-source framework [11].

This report first outlines the development of *Chloe*, an information and self-assessment tool to respond to a rapidly evolving health crisis. This was achieved by incrementally leveraging AI tools in order to address the challenges associated with the volume of demands and constantly changing information during the early phases of the COVID-19 pandemic. This paper then highlights *Chloe*’s strengths, the challenges we faced, and future directions for chatbot development to support infodemic management.

We wish to note that *Chloe* was designed and launched in an emergency effort to support public health authorities in keeping Canadians informed during the first wave of the COVID-19 pandemic before any governmental information tools had been deployed. Following the rollout of official and local public health online self-assessment tools providing features similar to ours, *Chloe* stopped being regularly updated.

## Chloe’s Development: From a Frequently Asked Questions Page to an Intelligent Chatbot

Increased uncertainty surrounding the previously unknown virus and disease resulted in a deluge of questions not only for our own care team but also for the entire Canadian health care system. In response to this surge, our Clinical Quality Committee led efforts to provide official answers to the most frequently asked questions (FAQs) from members on our care platform. This led to the release of the first version of our FAQ section on our website on March 5, 2020.

An FAQ section addresses predictable queries, but the constant flow of new information associated with the COVID-19 pandemic required a dynamic tool. In response to this challenge, we designed a self-assessment flow based on official available medical information surveyed by our care team. This allowed us to efficiently sort through and respond to the diverse concerns our members and clients had about COVID-19. We then created a smart virtual medical assistant that we named *Chloe for COVID-19* that was released publicly for all Canadians on March 9, 2020. In accordance with Dialogue’s rigorous data security and privacy policies, every effort was made to ensure that Canadians could use *Chloe* in an anonymous and secure fashion. To that effect, no information that could help identify the user (including but not limited to IP addresses and approximate location) was collected by default.

**Figure 1:**
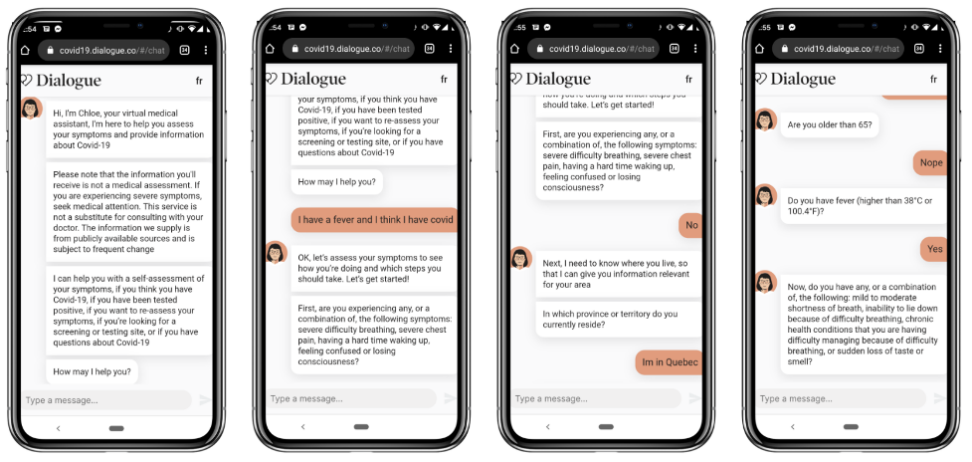
Example of self-assessment flow for a user in Quebec.

*Chloe* would ask a user about their symptoms, location, travel history, and recent contacts. This assessment resulted in a personalized recommendation including links to local resources such as the user’s provincial government’s COVID-19 guidelines. Figure 1 presents one example of the start of the assessment flow for a user in Quebec.

When provincial online self-assessment tools were later deployed, we integrated this information into *Chloe*. Our self-assessment tool saw the highest traffic on March 15, 2020, with 5945 new users accessing the tool, coinciding with the overflow of public health information phone lines. By April 18, 2020, it had been used 73,000 times, further indicating *Chloe’s* contribution to overall public health efforts in keeping Canadians informed.

In order to keep up with constantly evolving information spanning multiple Canadian jurisdictions and to discriminate between official information and disinformation, we designed and built a web scraper. This automated service was programmed to extract the latest data from reliable sources identified by our medical team. During the peak of the first wave, it ran every hour. When the flow of new information later slowed, it was then activated every 4 hours to ensure *Chloe* as well as our online FAQ still offered the most up-to-date information to Canadians.

In addition to the self-assessment tool and FAQ, we developed a question and answer (Q&A) model within *Chloe* to further meet the information needs of Canadians regarding COVID-19. The up-to-date COVID-19 information identified by our web scraper was used to refine the Q&A model, which was then further optimized using machine learning techniques. Our team of applied researchers developed a machine learning model that would find the correct answer to a given question about COVID-19 by feeding it approximately 4000 questions acquired through crowdsourcing and several thousand more from data collection tools offered by Amazon Web Services [12].

Given the time-consuming nature of this process and the need to gain a better understanding of what questions were most commonly asked, we used clustering to divide the approximately 4000 questions into 40 groups of similar questions. Clustering is a form of unsupervised machine learning, which aims to segregate groups with similar traits and assign them into clusters. The clustering algorithm used word embeddings to represent each of the questions in a multidimensional way. We were then able to accurately measure the similarity among the questions and split them into groups accordingly. For instance, all questions asking about how long the coronavirus could survive on surfaces, one of our most commonly asked questions, were grouped into one cluster.

**Figure 2:**
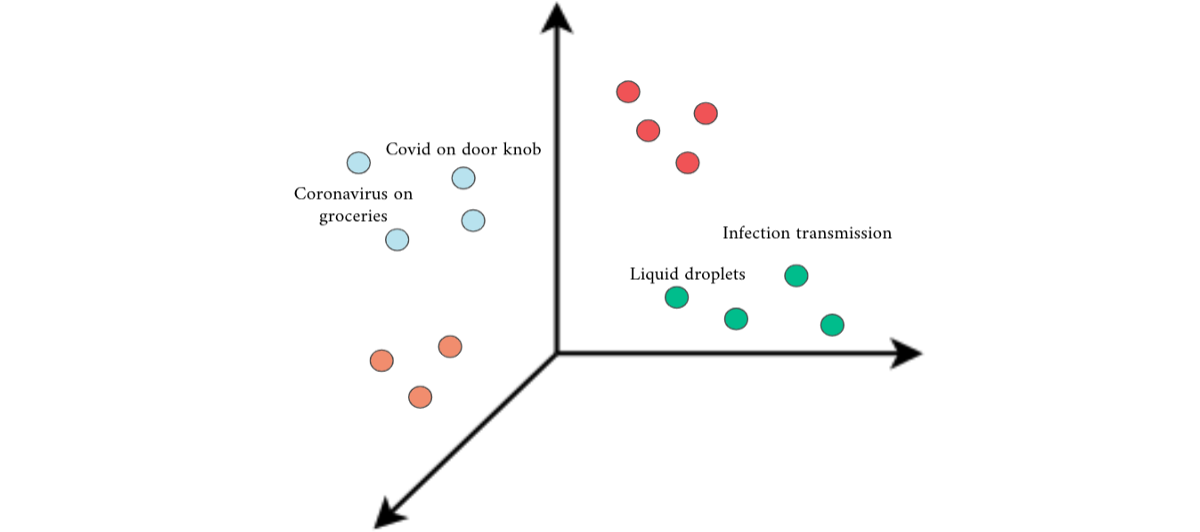
Representation of questions in a multidimensional space.

On April 28, 2020, we released our first interactive Q&A model, marking our transition from an FAQ system to a customized conversational experience. As a first iteration of a natural language Q&A model, we implemented a distance-computing, similarity-matching algorithm. In this model, all clustered questions were embedded in a multidimensional space. Figure 2 represents this step of the process, in which each colored circle is a question and its position is determined by the attributes computed by the algorithm. Averaging techniques were used to obtain the center of each cluster. This identified the average features of the questions within the same cluster.

For example, it was determined that the average question regarding the coronavirus on surfaces holds a combination of the word “surface” or “object” and a declension of the word “time.” Next, we embedded the user’s question using the same pretrained model, to have it in the same multidimensional space as the cluster averages. The distance between a user’s question and the clusters could be computed mathematically. The closest cluster could then be identified and the question categorized.

For users’ questions that matched a related cluster, we could then return the associated Q&A. For instance, the question “How long does it take for coronavirus to vanish from doorknobs?” was matched to the cluster that dealt with the virus on surfaces. We could then return the associated question-answer pair that would look like this:

How long can coronavirus stay on surfaces? It has been demonstrated that it can last for X hours on Y surface.

If the distance between the user’s question and each of the cluster’s centers was too great, however, the question would not be categorized, and an answer was not returned. In such cases, the user would be offered a link to our FAQ page, and *Chloe* would ask the user if they had another question. Our monitoring team also took note of these situations to further train *Chloe* to be able to answer an increasing number of user questions. This was achieved by having our team analyze these additional questions and add them to the set of questions used to retrain the clustering algorithm.

Canada being a bilingual country, we also needed this model to accurately return question-answer pairs to all Canadians in both English and French. We therefore followed the aforementioned process twice in order to cover questions in both languages.

**Figure 3:**
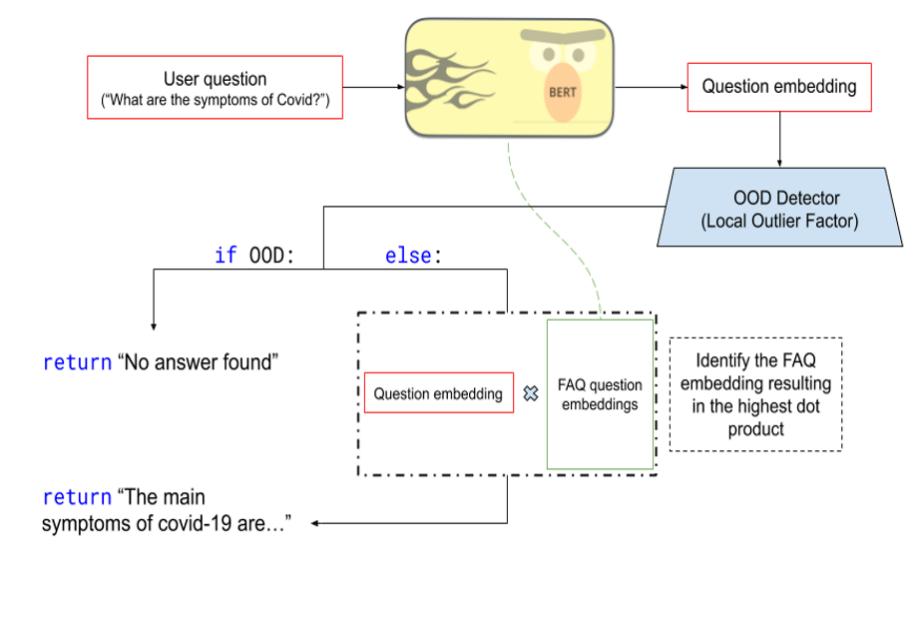
Diagram of the final question & answer (Q&A) model architecture using the example of a question about the symptoms of COVID-19. BERT: Bidirectional Encoder Representations from Transformers; FAQ: frequently asked question; OOD: out-of-distribution.

While developing the previously described similarity model, we also worked on a more advanced model consisting of 2 main components: a Bidirectional Encoder Representations from Transformers (BERT) model [13] that ranked all COVID-19 FAQ questions from trusted sources according to their relevance with a user’s question and an out-of-distribution (OOD) detector using a Local Outlier Factor algorithm [14]. The purpose of this OOD detector was to eliminate questions that could not be answered with the available information from the vetted sources the chatbot used. This improved the number of questions answered and the accuracy of the answers provided while minimizing manual updating work from our care team. Figure 3 presents a general diagram of the model architecture.

Table 1 describes the results obtained from this final Q&A model’s accuracy. Overall accuracy represents the model’s ability to give a correct answer to the user’s question, and OOD accuracy represents its ability to detect situations in which the question asked has no answer in our data sources. The BERT pretrained model we used has a better performance in English, which explains the discrepancy between the English and French results. Although higher metrics can be obtained from Q&A models, we believe that these results are reasonable given time and data constraints. These results were also deemed acceptable because our Q&A model sought to provide answers to commonly asked questions and not directly inform patient care.

**Table 1:** Overall and out-of-distribution (OOD) accuracies of the final question & answer (Q&A) model in English and in French.

Language	Overall accuracy, %	OOD accuracy, %
English	81.83	90.48
French	71.32	70.60

We nevertheless addressed the error rates of our model to the best of our abilities by consistently displaying the question and answer as a pair when responding to the user. For example, if the user asked about coronavirus and pets and *Chloe* replied with information about the coronavirus and surfaces, the user would know that the model was responding about surfaces and not pets. This ensured the user would not be misled by false information. Lastly, the model was validated using a standard process [15]. The dataset was divided into training, validation, and test sets, followed by an evaluation of how the test set (not previously seen by the model) performed. Further details about the Q&A model are detailed in a poster presented at the Montreal AI Symposium 2020 [16].

Two other important features were implemented to increase *Chloe’s* ability to meet the evolving needs of Canadians: a daily self-health assessment and COVID-19 testing navigation. The self-health check feature was added to the assessment flow at the end of May 2020. Users choosing to enroll in this feature received a daily SMS message with a link to their self-health check-in and had the choice to opt out at any time. Anyone using this self-health assessment service had their first name and phone numbers securely stored on our data servers located in Canada. Having regular contact with users helped tailor the assessment to the user’s particular situation and needs. When starting the assessment flow, users’ previously reported symptoms and concerns helped the chatbot identify relevant questions to be asked and offer appropriate recommendations. For example, if the user reported a fever the day before, the chatbot might ask if it was still present and if it had gotten better or worse.

**Figure 4:**
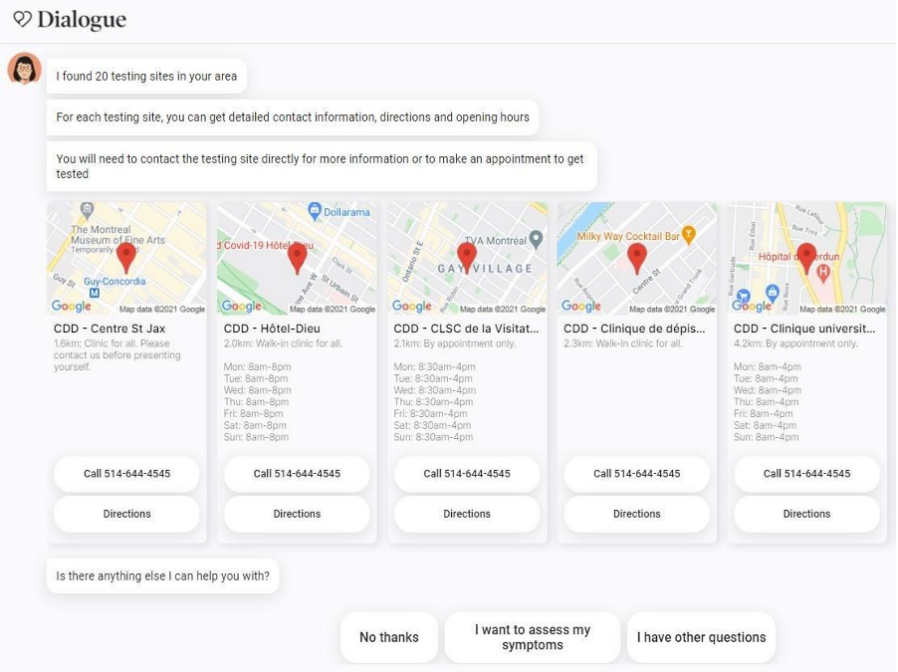
Example of test navigation results for a user in Quebec.

The testing navigation feature was added on June 2, 2020 to the assessment flow to support public health officials and public health information phone lines. This feature was built in partnership with Clinia [17], a technology company enabling health care resource navigation. By integrating Clinia’s application programming interface into *Chloe*, any user whose self-assessment resulted in a recommendation to get COVID-19 testing would be prompted with the option to search for testing sites. Once the user entered their desired location to get tested, they would receive a list of sites offering COVID-19 testing, their opening hours, special instructions, as well as a link to obtain directions to the sites. Figure 4 presents an example of local testing sites offered to a user in Quebec seeking navigation assistance.

**Figure 5:**
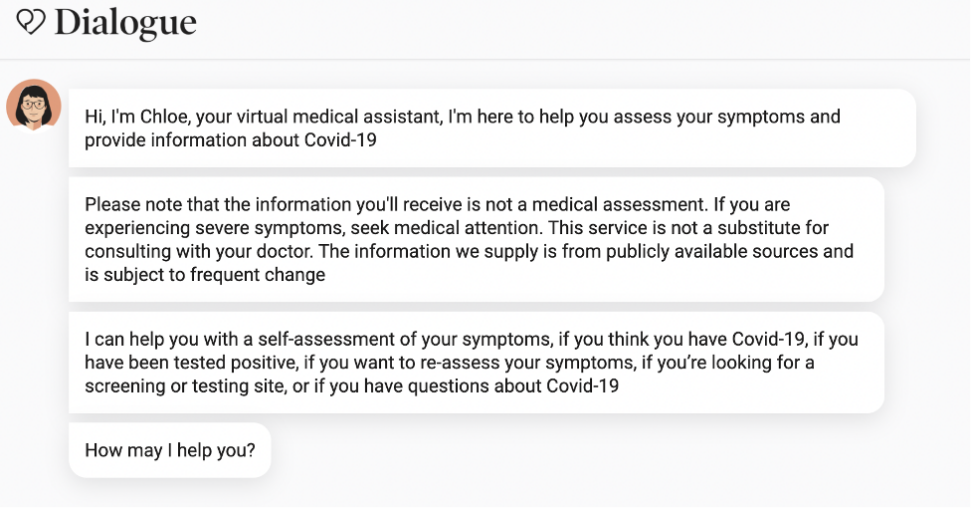
*Chloe’s* welcome message to users explaining their options.

**Figure 6:**
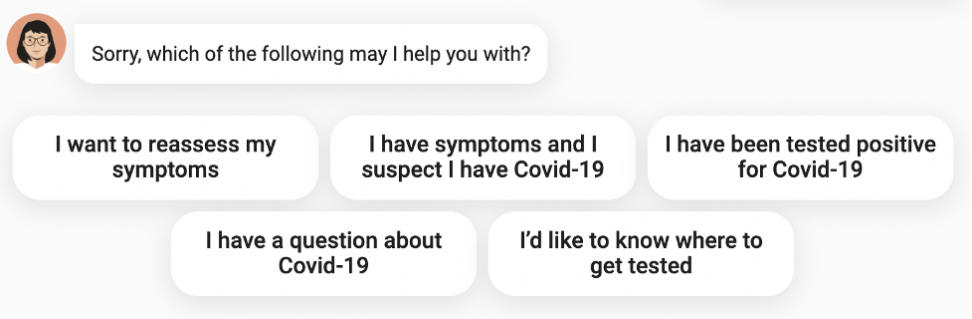
Display of *Chloe’s* features available to users.

To make the conversation feel as natural as possible for users, we leveraged a machine-learning technology called natural language understanding (NLU), a subtopic of natural language processing, which converts human-readable text inputs into machine language that can be processed to extract an understanding. In our particular case, we extracted intents, which represent the user’s query, and entities, which represent key categorized values. For example, if a user said, “I want to find a test site close to Montreal,” NLU would help us map the user’s intent to something like “find_test_site” and extract the entity “Montreal,” which we know is a city. Test sites in Montreal could then easily be suggested to the user. The use of NLU allowed for more open-ended questions akin to normal conversations and minimized clicking. As of June 29, 2020, *Chloe* could ask users: “How may I help you?” NLU components were associated with each possible question, providing a natural conversational experience with an intelligent chatbot. In terms of dialogue management strategies, we used a combination of NLU and static flows defined by a multidisciplinary team of medical experts, software and machine learning engineers, and conversational designers. Figure 5 presents what the latest version of *Chloe* looks like to users upon first connecting with the chatbot. In response to *Chloe*’s initial “How may I help you?”, if a user’s response falls outside the clusters of questions known to *Chloe*, the chatbot presents 5 clear clickable options from which the user can choose. Figure 6 shows *Chloe’s* main features as presented to users.

## Strengths, Challenges, and Future Directions

To our knowledge, at the onset of the COVID-19 pandemic, there were no other accessible chatbots that provided free, local, up-to-date information on COVID-19 in both English and French to the Canadian public. As of early March 2020, *Chloe for COVID-19* filled this important gap by supporting efforts in keeping Canadians informed when public health hotlines were most overwhelmed. *Chloe* underwent rigorous training by a dedicated team of medical and technology experts who were involved from inception to deployment. This team continually monitored the chatbot using analytics data, making adjustments and updates when needed. Informal user testing was conducted each time a major update was introduced. This involved approximately 10 individuals recruited internally who tested out the different available flows and provided feedback.

As the pandemic unfolded and more information emerged about COVID-19, *Chloe* adapted to address evolving questions from the public. In her most advanced version in June 2020, *Chloe* uniquely integrated 5 features into 1 bilingual chatbot: a self-assessment tool, resources for users who tested positive for COVID-19, answers to common questions about COVID-19, testing navigation, and a daily self-health check-in.

Despite *Chloe’s* success in providing tailored information to the Canadian public early on in the pandemic, we encountered several challenges during *Chloe’s* development and after *Chloe’s* launch, of which the most notable were keeping up with the increasingly vast and rapidly changing information available on COVID-19 as well as time and data constraints within an emergency health crisis context.

Rapidly changing information on COVID-19 (eg, how the disease presents in individuals, how the SARS-CoV-2 virus is transmitted, and when individuals should get tested), complicated by Canada’s multijurisdictional landscape with guidelines changing from one region to another, required constant monitoring from our team to ensure accurate information was provided to users across the country. Furthermore, as mentioned earlier in this report, chatbots have the potential to address the questions of millions of people at once [4], but the risk of amplifying misinformation and the lack of research on chatbot effectiveness present significant challenges, particularly in a pandemic context [7]. By reinforcing local provincial and territorial guidelines, we ensured that *Chloe’s* messaging to users aligned with the most up-to-date public health information tailored to each user’s location and health situation. We learned that having a multidisciplinary team of technology, nursing, physician, customer success, and clinical quality experts, assisted by AI tools, was the best approach to quickly mobilize all necessary information and make any required adjustments to *Chloe* as the pandemic situation evolved.

Given that we developed *Chloe* as an informational tool in an emergency effort to support overwhelmed public health hotlines, we lacked the time and resources needed to rigorously evaluate *Chloe*’s implementation and measure *Chloe’s* impact on users. In a noncrisis context, it would have been ideal to set and track specific implementation metrics other than general chatbot traffic to improve *Chloe* more systematically. In this instance, we prioritized the need to act quickly in assisting public health efforts at the height of a health crisis. We also considered that implementation metrics are particularly important for chatbots that directly inform patient care, such as those that refer users to consultations with health professionals. Those chatbots require closer monitoring than chatbots such as *Chloe*, whose main goal was to reliably answer the public’s questions about COVID-19 and to redirect individuals to testing services and local public health guidelines when needed.

Moreover, we were unable to precisely measure *Chloe’s* acceptability, but we acknowledge the importance of evaluating this metric [18]. Current metrics used to determine the acceptability of AI chatbots in health care include trustworthiness of the information provided as well as a user experience that is perceived as empathetic to the patient [19]. Despite not formally evaluating *Chloe’s* acceptability, *Chloe* was designed to be trustworthy, by offering up-to-date official information from public health sources, and empathetic, by leveraging AI techniques to make the conversation feel as natural as possible with users. We also knew from prior experience designing and implementing health technology tools for the Dialogue platform, as well as extensive member feedback, that chatbots are an acceptable and easy-to-use tool for collecting, assessing, and sharing health information. That being said, rigorous user testing combined with the systematic collection of user feedback, for instance through the integration of a permanent feedback link in the user interface of a chatbot as done by Herriman et al [4], may have allowed us to better understand user needs and make quicker improvements to *Chloe’s* features.

In further building on *Chloe’s* development and envisioning the future of chatbots, we would also prioritize evaluating the impact of chatbots on how well individuals feel informed. In a scoping review of the technical metrics used to evaluate healthcare chatbots, Abd-Alrazaq et al [20] found that most chatbots were assessed according to 27 different metrics, with global usability and survey designs as the most commonly used metrics. Although chatbots like *Chloe* are not “healthcare chatbots” in that they do not deliver healthcare services, the evaluation of their usability and performance is warranted in order to improve their ability to provide the right information to users at the right time.

In summary, we believe *Chloe’s* development is useful in illustrating the potential for rapid collaboration among health, technology, and corporate sectors to assist public health efforts in keeping individuals informed at the height of a health crisis. Addressing the public’s need for accurate health information will remain important within the current pandemic context and during any future health crises. As COVID-19 vaccination campaigns continue to target the general public, intelligent conversational agents have the potential to play a critical role in debunking misinformation surrounding vaccines and directing individuals to public health resources [21,22]. One study in the preprint stage, which examined a chatbot designed to address arguments against the COVID-19 vaccine, promisingly showed that individuals’ attitudes towards vaccination became more positive after receiving information from this chatbot [23]. Moreover, in a recent Nature survey of infectious disease researchers, virologists, and immunologists in 23 countries, the majority of respondents predicted that the SARS-CoV-2 virus will likely become endemic over time, with its future closely depending on virus mutations and the type of immunity individuals will acquire [24]. In anticipation of the upcoming waves of uncertainty regarding this virus in the coming years, we envision an ongoing and growing role for chatbots in supporting public health efforts in infodemic management in order to keep the public safe and informed.

## Conclusion

The devastating and widespread health consequences of the still ongoing COVID-19 pandemic and continued uncertainty surrounding this novel disease are unprecedented challenges to public health. Since the start of the pandemic, managing the ensuing deluge of both information and misinformation about COVID-19 has also required unparalleled efforts from health authorities.

In detailing the development of *Chloe for COVID-19*, we provide an encouraging example of collaboration leading to a rapidly scalable, safe, secure, and useful human-enhanced digital tool to address the information needs of Canadians during this pandemic. Continuing to leverage the potential of such chatbots to become trusted public health allies will support ongoing infodemic management strategies. As research on the safety and acceptability of these tools advances, we anticipate a growing role for chatbots, both standalone and integrated with human-provided care, in the current pandemic, in particular with vaccination rollout and ongoing efforts to combat misinformation.

## Abbreviations

AI: artificial intelligence
BERT: Bidirectional Encoder Representations from Transformers
FAQ: frequently asked questions
NLU: natural language understanding
OOD: out-of-distribution
Q&A: question and answer

## References

[1] Vraga EK, Jacobsen KH (2020). Strategies for Effective Health Communication during the Coronavirus Pandemic and Future Emerging Infectious Disease Events. World Medical & Health Policy.

[2] (2021). Infodemic Management. World Health Organization.

[3] (2020). Info-Santé 811 ne suffit pas à la demande. Radio Canada.

[4] Herriman M, Meer E, Rosin R, Lee V, Washington V, Volpp KG (2020). Asked and answered: Building a chatbot to address covid-19-related concerns. NEJM Catalyst Innovations in Care Delivery.

[5] Abdul-Kader SA, Woods JC (2015). Survey on Chatbot Design Techniques in Speech Conversation Systems. International Journal of Advanced Computer Science and Applications.

[6] Dale R (2016). The return of the chatbots. Nat. Lang. Eng.

[7] Miner AS, Laranjo L, Kocaballi AB (2020). Chatbots in the fight against the COVID-19 pandemic. NPJ Digit Med.

[8] Chloe. Dialogue Health Technologies.

[9] Liu J, Gao Z, Kang Y, Jiang Z, He G, Sun C, Liu X, Lu W (2021). Time to Transfer: Predicting and Evaluating Machine-Human Chatting Handoff. AAAI.

[10] Harms J, Kucherbaev P, Bozzon A, Houben G (2019). Approaches for Dialog Management in Conversational Agents. IEEE Internet Comput.

[11] (2021). Rasa.

[12] Amazon Mechanical Turk.

[13] Devlin J, Chang M, Lee K, Toutanova K (2019). Bert: Pre-training of deep bidirectional transformers for language understanding. Cornell University.

[14] Breunig M, Kriegel H, Ng R, Sander J (2000). LOF. SIGMOD Rec.

[15] Brownlee J (2020). What is the Difference Between Test and Validation Datasets?. Machine Learning Mastery.

[16] Bronzi M, Pinto J, Ghosn J, Subakan C, Sharma P, Lu X (2020). Poster 58: A Question Answering System in Response to the COVID-19 Crisis. https://drive.google.com/file/d/1chJyp6mEhieL13EzbX_Xpf8m3MenDuDE/view?pli=1.

[17] Clinia.

[18] Tudor Car L, Dhinagaran DA, Kyaw BM, Kowatsch T, Joty S, Theng Y, Atun R (2020). Conversational Agents in Health Care: Scoping Review and Conceptual Analysis. J Med Internet Res.

[19] Nadarzynski T, Miles O, Cowie A, Ridge D (2019). Acceptability of artificial intelligence (AI)-led chatbot services in healthcare: A mixed-methods study. Digit Health.

[20] Abd-Alrazaq AA, Rababeh A, Alajlani M, Bewick BM, Househ M (2020). Effectiveness and Safety of Using Chatbots to Improve Mental Health: Systematic Review and Meta-Analysis. J Med Internet Res.

[21] Vedhara K (2021). Experts create ‘chatbot’ to address people’s concerns about Covid-19 vaccines. University of Nottingham.

[22] Kinsella B (2020). Nuance Launches COVID-19 Vaccine Bot and Vaccine Assistant. Voicebot.ai.

[23] Altay S, Hacquin A, Chevallier C, Mercier H (2021). Information Delivered by a Chatbot Has a Positive Impact on COVID-19 Vaccines Attitudes and Intentions. PsyArXiv.

[24] Phillips N (2021). The coronavirus is here to stay - here's what that means. Nature.

